# Supraphysiological Dose of Testosterone Impairs the Expression and Distribution of Sex Steroid Receptors during Endometrial Receptivity Development in Female Sprague–Dawley Rats

**DOI:** 10.3390/ijms251810202

**Published:** 2024-09-23

**Authors:** Allia Najmie Muhammad Yusuf, Mohd Fariz Amri, Azizah Ugusman, Adila A Hamid, Mohd Helmy Mokhtar

**Affiliations:** 1 Department of Physiology, Faculty of Medicine, Universiti Kebangsaan Malaysia, Cheras, Kuala Lumpur 56000, Malaysia; allia.najmie@ums.edu.my (A.N.M.Y.); dr.azizah@ppukm.ukm.edu.my (A.U.); adilahamid@ppukm.ukm.edu.my (A.A.H.); 2 Department of Biomedical Sciences, Faculty of Medicine and Health Sciences, Universiti Malaysia Sabah, Kota Kinabalu 88400, Malaysia; 3 Department of Pathology and Microbiology, Faculty of Medicine and Health Sciences, Universiti Malaysia Sabah, Kota Kinabalu 88400, Malaysia; fariz@ums.edu.my

**Keywords:** testosterone, endometrial receptivity, sex steroid receptors, androgen, progesterone, oestrogen

## Abstract

This study aims to investigate the effect of a supraphysiological dose of testosterone on the levels of sex steroid hormones and the expression and distribution of sex steroid receptors in the uterus during the endometrial receptivity development period. In this study, adult female Sprague–Dawley rats (*n* = 24) were subcutaneously administered 1 mg/kg/day of testosterone alone or in combination with the inhibitors (finasteride or anastrozole or both) from day 1 to day 3 post-coitus, while a group of six untreated rats served as a control group. The rats were sacrificed on the evening of post-coital day 4 of to measure sex steroid hormone levels by ELISA. Meanwhile, gene expression and protein distribution of sex steroid receptors were analysed by quantitative polymerase chain reaction (qPCR) and immunohistochemistry (IHC), respectively. In this study, treatment with a supraphysiological dose of testosterone led to a significant reduction in oestrogen and progesterone levels compared to the control. The mRNA expression of the androgen receptor increased significantly in all treatment groups, while the mRNA expression of both the progesterone receptor and the oestrogen receptor-α decreased significantly in all treatment groups. The IHC findings of all sex steroid receptors were coherent with all mRNAs involved. This study shows that a supraphysiological dose of testosterone was able to interrupt the short period of the implantation window. This finding could serve as a basis for understanding the role of testosterone in endometrial receptivity in order to develop further therapeutic approaches targeting androgen-mediated disorders of endometrial receptivity.

## 1. Introduction

Testosterone is a male sex steroid hormone that is also found in women, albeit in smaller quantities than in men [1,2]. The importance of oestrogen and progesterone for female reproduction is well documented, but the role of testosterone in women’s reproductive health is less emphasised [2]. In women of reproductive age, serum androgen levels are generally higher than oestrogen levels, although oestrogens are considered the primary sex steroid hormone. An exception is the pre-ovulatory and mid-luteal phase of the menstrual cycle, when androgen and oestrogen levels are comparable [3,4]. Approximately 0.02 µmol of testosterone is secreted per day by the theca cells of the ovaries and the adrenal cortex [5]. The peripheral conversion of androstenedione (prohormone) in adipose tissue is responsible for about half of the circulating testosterone [6]. In order for prohormones such as dehydroepiandrosterone sulphate (DHEAS), dehydroepiandrosterone (DHEA) and androstenedione to activate the androgen receptors, they must first be converted into testosterone and dihydrotestosterone (DHT) [7].

Androgen production in the ovaries and the conversion of testosterone to oestradiol are essential for the physiological process of ovulation, and insufficient androgen production in the follicular phase can lead to anovulation [8,9]. Research on the role of androgens in women has focused almost exclusively on issues of excess [10]. Much of the evidence is based on extrapolation of data from in vitro and animal studies [3]. However, despite extensive research, only a few causes of androgen excess in women are known. The most widely discussed or researched cause is polycystic ovary syndrome (PCOS) and the increasing concern over anabolic and androgenic steroid (AAS) use in female athletes, which has been linked to women’s reproductive health. PCOS is thought to be caused partly by abnormal ovarian steroidogenesis leading to functional ovarian hyperandrogenism [11]. Increased secretion of primary androgens by theca cells of polycystic ovaries, together with increased drive by luteinising hormone (LH), contributes to increased ovarian androstenedione and testosterone production in the ovaries [12,13]. Meanwhile, the use of supra-physiological AASs is widespread as they can increase muscle growth to improve the performance of athletes or for aesthetic purposes with minimal androgenic effects. Long-term use of AASs leads to side effects that affect major organs such as the reproductive organs and the cardiovascular system [14]. The most common side effects associated with the female reproductive system are breast atrophy, clitoral hypertrophy, menstrual irregularities and uterine atrophy [15].

In the meantime, understanding endometrial receptivity is crucial for explaining unexplained infertility and pregnancy loss. Endometrial receptivity refers to the brief window of endometrial maturation during which the trophectoderm of the blastocyst is able to adhere to endometrial epithelial cells before invading the endometrial stroma and vasculature [16,17]. The increase in oestrogen hormone during the follicular phase helps to promote the proliferation of the endometrium and thus increase the sensitivity of the endometrium to oestrogen and progesterone by increasing the levels of oestrogen receptor alpha (ERα) and progesterone receptor (PR). Following ovulation, progesterone is secreted by the luteinised follicles, which leads to differentiation of the endometrial cells. This is evidenced by the segregated expression of the progesterone receptor (PR) and the oestrogen receptor (ER) in the peri-implantation phase, which sheds light on where the coordinated effects of progesterone and oestrogen lie in preparing the uterus for implantation and decidualisation in the early stages of pregnancy [18]. In addition, androgens regulate endometrial receptivity by binding to the androgen receptor (AR), which is widely distributed in the uterus particularly in its luminal epithelium, glands and stroma [19].

However, information on the effects of androgen excess or hyperandrogenism, as seen in PCOS and the use of AASs in women during the development of endometrial receptivity is still limited and requires further investigation. Therefore, in this study we investigate the adverse effects of a supraphysiological dose of testosterone on the development of endometrial receptivity in terms of sex steroid receptor expression and distribution to understand the problems of infertility in patients with high androgen concentrations. This will also provide a basis for the development of future treatments that can improve pregnancy outcomes in women with hyperandrogenism.

## 2. Results

### 2.1. Effects of Supraphysiological Dose of Testosterone on Serum Levels of Sex Steroid Hormones

**Figure 1:**
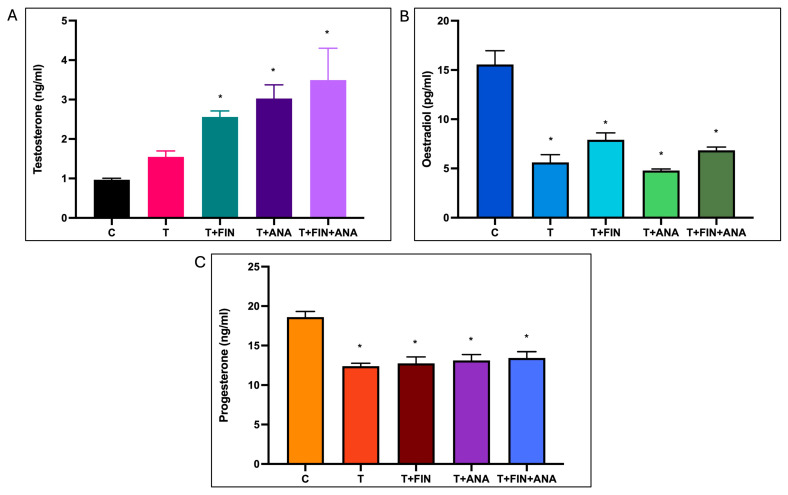
Effects of testosterone on serum levels of (**A**) testosterone, (**B**) oestradiol, (**C**) progesterone. C: normal control; T: testosterone propionate; T + FIN: testosterone propionate + finasteride; T + ANA: testosterone propionate + anastrozole; T + FIN + ANA: testosterone propionate + finasteride + anastrozole. Error bars represent standard error of the mean (SEM). *n* = 6 in each group, * *p* < 0.05 significant compared to normal control group.

Figure 1A shows the testosterone level in each group. The testosterone level increases steadily in the trend. The bar graph shows that testosterone levels increased significantly in the rats treated with the combination testosterone + finasteride, the combination testosterone + anastrozole and the combination testosterone + finasteride + anastrozole compared to the normal control rats.

Meanwhile, Figure 1B shows the effect of testosterone on serum oestradiol levels. Oestradiol levels were significantly reduced in testosterone-treated rats compared to normal control rats. The oestradiol level was significantly lower in the testosterone group compared to the normal control rats. The significant reduction in oestradiol level (*p* < 0.05) was demonstrated in rats treated with the combination of testosterone + finasteride, the combination of testosterone + anastrozole and the combination of testosterone + finasteride + anastrozole, with the combination of testosterone + anastrozole having the lowest oestradiol level among the combinations.

As can be seen in Figure 1C, the control group again had the highest average serum progesterone level at around 18 ng/mL. Progesterone levels were significantly reduced in the testosterone-treated rats compared to the normal control group. Treatment with the combination of testosterone + finasteride, the combination of testosterone + anastrozole or the combination of testosterone + finasteride + anastrozole significantly reduced progesterone levels compared to the normal control rats (*p* < 0.05). The reduction in progesterone levels appears to be less pronounced than that of oestradiol, with the testosterone + anastrozole and testosterone + finasteride + anastrozole groups showing relatively higher progesterone levels than the testosterone alone and testosterone + finasteride groups.

### 2.2. Effects of Supraphysiological Dose of Testosterone on Protein Distribution and mRNA Expression of Androgen Receptor (AR)

**Figure 2:**
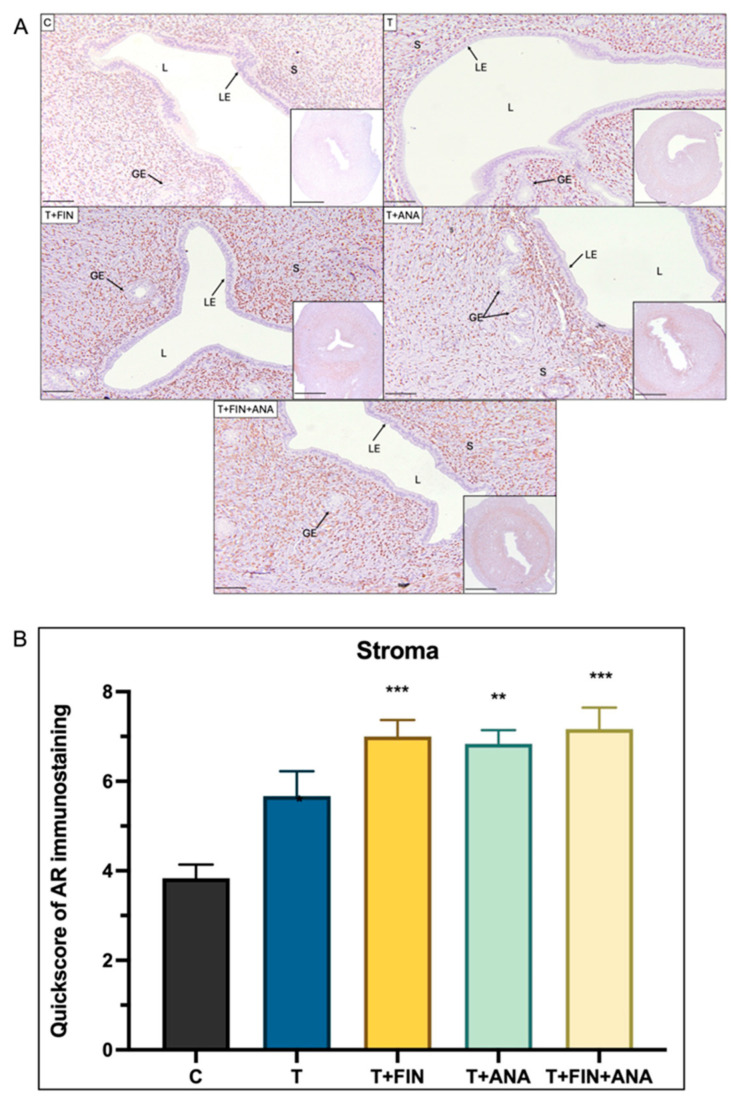
Distribution of the androgen receptor (AR) in the uterus of rats. (**A**) The dark brown colouring, indicated by arrows, indicates the antibody binding site of the AR, which appears to be located in the stroma. Strong dark brown staining was observed in all groups only in the stroma, but not in the epithelial gland and the glandular epithelium. (**B**) Semi-quantitative evaluation of AR immunostaining for the stroma. C: normal control; T: testosterone propionate; T + FIN: testosterone propionate + finasteride; T + ANA: testosterone propionate + anastrozole; T + FIN + ANA: testosterone propionate + finasteride + anastrozole. LE: luminal epithelium; GE: glandular epithelium; L: endometrial lumen; S: stroma. Scale bar = 200 μm, scale bar inset = 1 mm, magnification 40× and 200×. Error bars represent standard error of the mean (SEM). *n* = 6 in each group, ** *p* < 0.01, *** *p* < 0.001 significance compared to normal control group.

Figure 2 shows the immunohistochemical analysis of the androgen receptor (AR) and its quickscore (QS) scoring analysis in the uterine stroma of rats in five different experimental groups. The control group serves as a baseline with an average QS for AR immunostaining around 3 (score range from 3 to 4). This value was derived from the components of the QS, which include the scoring for proportion (0–5) and scoring for intensity (0–3) with a maximum total score of 8. Treatment with testosterone alone appears to significantly increase the QS value for AR immunostaining to just above 5 (*p* < 0.05). In addition, the QS value remains significantly increased in the combination of testosterone + finasteride group compared to the control group (*p* < 0.001). The combination of testosterone + anastrozole group also showed a similar trend to the testosterone and the combination of testosterone + finasteride groups (*p* < 0.01) with a significant increase in QS compared to the control group. Similar to the combination of testosterone + finasteride, the group with the combination of both drugs also showed a highly significant increase in AR in the stroma (*p* < 0.001).

**Figure 3:**
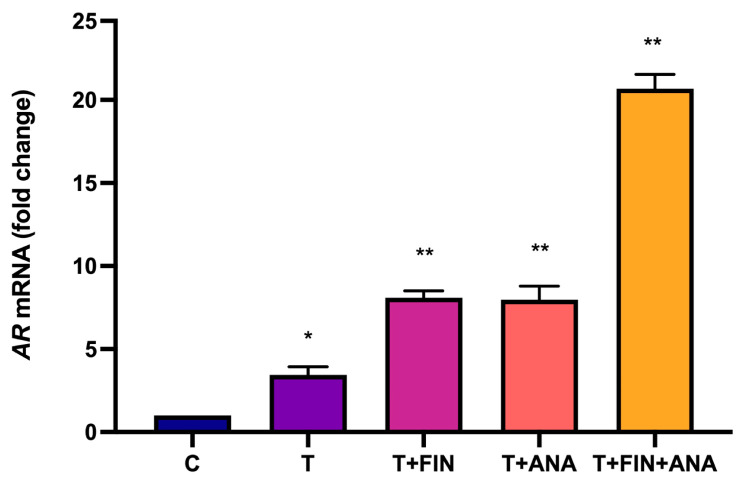
Effects of testosterone on androgen receptor (AR) mRNA expression. C: normal control; T: testosterone propionate; T + FIN: testosterone propionate + finasteride; T + ANA: testosterone propionate + anastrozole; T + FIN + ANA: testosterone propionate + finasteride + anastrozole. Error bars represent standard error of the mean (SEM). *n* = 6 in each group, * *p* < 0.05, ** *p* < 0.01 significant compared to the normal control group.

Figure 3 shows the change in mRNA expression of AR in response to different treatments with testosterone and its combinations with finasteride or anastrozole. In this study, treatment with testosterone resulted in a significant increase in AR mRNA expression (*p* < 0.05) compared to the control group. The same trend was observed with the combination of testosterone + finasteride and testosterone + anastrozole groups, where there was a significant increase in AR mRNA expression (*p* < 0.01) compared to the control group. Treatment with the combination of testosterone + finasteride + anastrozole also led to the highest significant increase in AR mRNA expression (*p* < 0.01) compared to the control group.

### 2.3. Effects of Supraphysiological Dose of Testosterone on Protein Distribution and mRNA Expression of Oestrogen Receptor-α (ERα)

**Figure 4:**
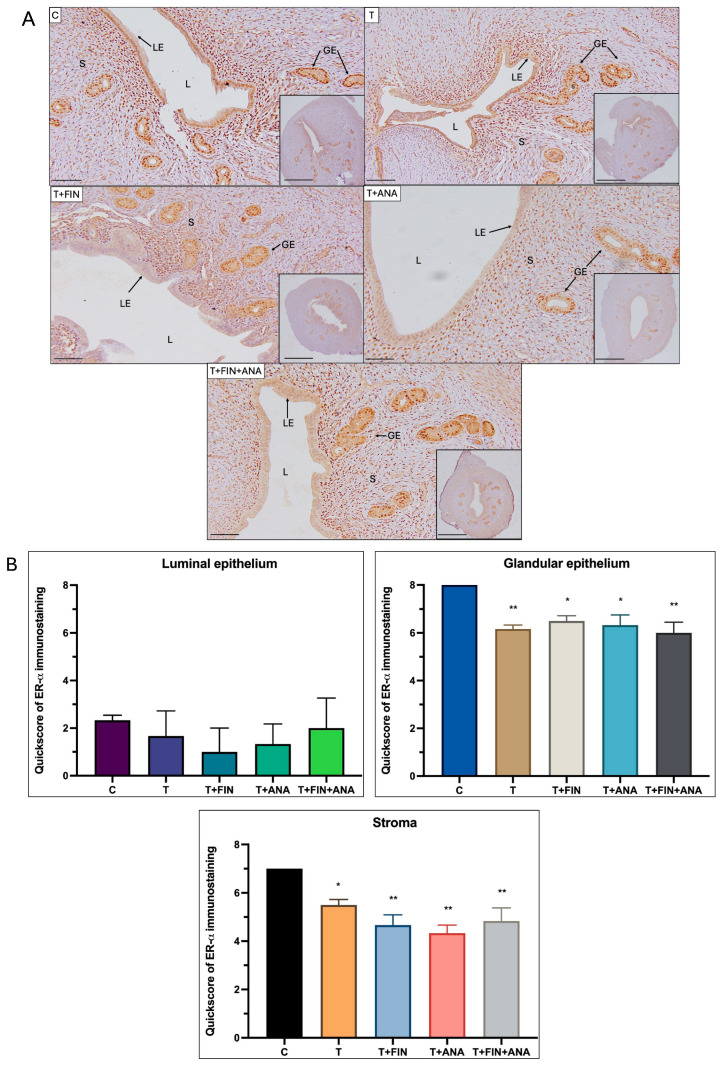
Distribution of oestrogen receptor-α (ERα) in the uterus of rats. (**A**) The dark brown colour, indicated by arrows, indicates the antibody-binding site of ERα, which appears to be present in the uterine lumen, uterine glandular epithelium and stroma. Strong dark brown staining was observed in the luminal epithelium in all four treatment groups compared to the control group, but to a lesser extent in the glandular epithelium and stroma. (**B**) Semi-quantitative scoring of ERα immunostaining for the endometrium compartments. C: normal control; T: testosterone propionate; T + FIN: testosterone propionate + finasteride; T + ANA: testosterone propionate + anastrozole; T + FIN + ANA: testosterone propionate + finasteride + anastrozole. LE: luminal epithelium; GE: glandular epithelium; L: endometrium lumen; S: stroma. Scale bar = 200 μm, scale bar inset = 1 mm, Magnification 40× and 200×. Error bars represent standard error of the mean (SEM). *n* = 6 in each group, * *p* < 0.05, ** *p* < 0.01 significance compared to normal control group.

Figure 4 reveals the quantification of ERα immunostaining in different compartments of the rat uterus: luminal epithelium, glandular epithelium and stroma. In the luminal epithelium, the group of normal rats shows a baseline QS for ERα staining. Another group of rats receiving testosterone treatment appears to decrease this score slightly, although not significantly. The combination of testosterone with finasteride and the combination of testosterone with anastrozole, both appear to further reduce QS, although not significantly. The same pattern is seen when both finasteride and anastrozole were combined with testosterone. The QS was reduced compared to the normal control group, although not significantly.

As for the glandular epithelium, the normal control group has a high ERα distribution. Treatment with testosterone significantly reduces ERα expression, as shown by the statistically significant reduction in QS (*p* < 0.01). Treatment with both testosterone + finasteride and testosterone + anastrozole also leads to a reduction in ERα expression, although the effect is less pronounced (*p* < 0.05). After treatment with the combination of testosterone + finasteride + anastrozole, the staining intensity of ERα was significantly lower compared to the control group (*p* < 0.01).

A similar trend can be observed in the stromal compartment. The normal control group has high ERα expression, and treatment with testosterone (*p* < 0.05) or testosterone + finasteride (*p* < 0.01) or testosterone + anastrozole (*p* < 0.01) or treatment of testosterone with both blockers’ finasteride and anastrozole (*p* < 0.01) significantly reduces the QS value.

**Figure 5:**
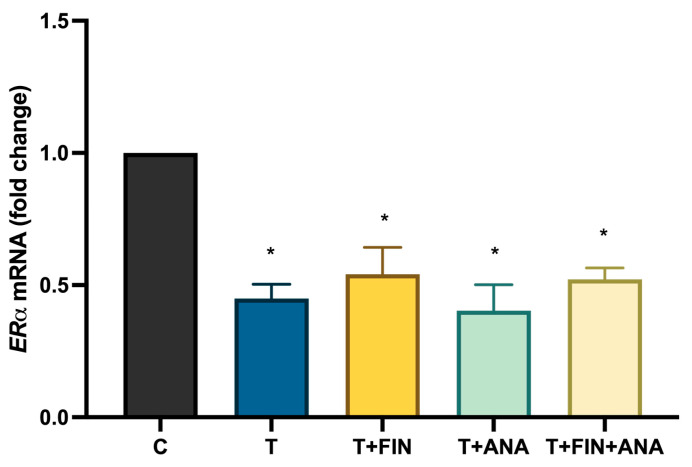
Effects of testosterone on oestrogen receptor alpha (ERα) mRNA expression. C: normal control; T: testosterone propionate; T + FIN: testosterone propionate + finasteride; T + ANA: testosterone propionate + anastrozole; T + FIN + ANA: testosterone propionate + finasteride + anastrozole. *n* = 6 in each group, * *p* < 0.05 significant compared to normal control group.

Figure 5 shows a bar chart illustrating ERα mRNA expression. Treatment with testosterone significantly (*p* < 0.05) decreases ERα mRNA expression compared to normal control rats. All treatments with the combination of testosterone + finasteride, testosterone + anastrozole and the combination of testosterone with both blockers continue to downregulate ERα mRNA expression while maintaining statistical significance (*p* < 0.05).

### 2.4. Effects of Supraphysiological Dose of Testosterone on Protein Distribution and mRNA Expression of Progesterone Receptor (PR)

**Figure 6:**
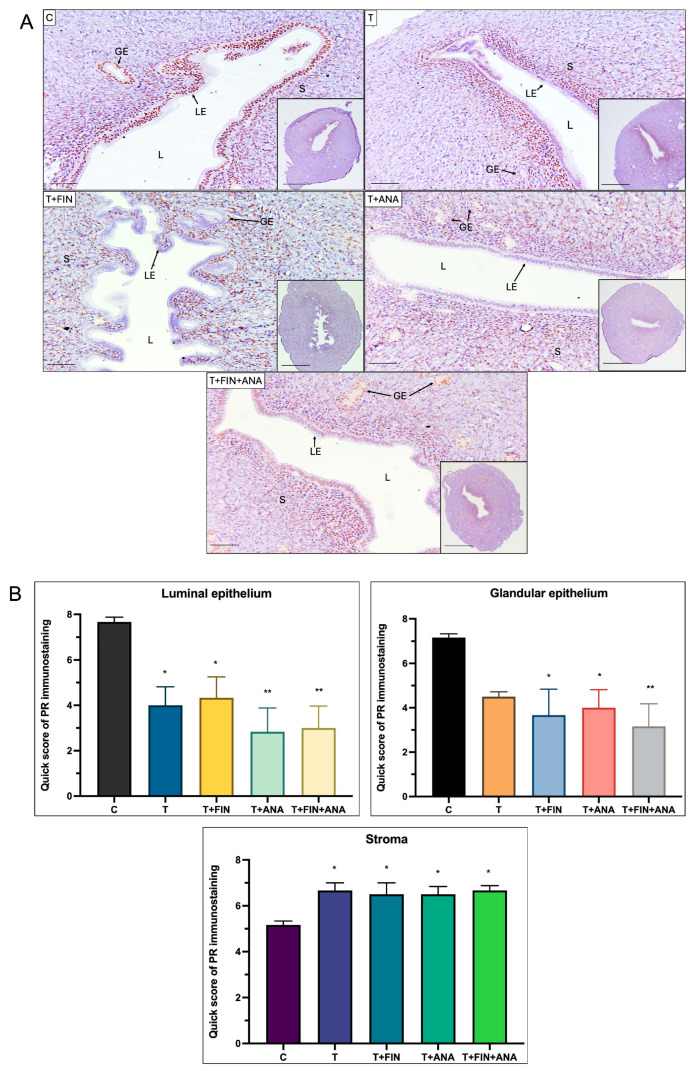
Distribution of progesterone receptor (PR) in the uterus of rats. (**A**) The dark brown colour indicated by arrows indicates the antibody binding site of PR, which appears to be present in the luminal and glandular epithelium of the uterus as well as in the stroma. In all four treatment groups, a strong dark brown colouration was observed in the stroma compared to the control group, but less so in the luminal and glandular epithelium. (**B**) Semi-quantitative evaluation of PR immunostaining for the endometrial compartments. C: normal control; T: testosterone propionate; T + FIN: testosterone propionate + finasteride; T + ANA: testosterone propionate + anastrozole; T + FIN + ANA: testosterone propionate + finasteride + anastrozole. LE: Luminal epithelium; GE: Glandular epithelium; L: Lumen of the endometrium; S: Stroma. Scale bar = 200 μm, scale bar insert = 1 mm, magnification 40× and 200×. Error bars represent standard error of the mean (SEM). *n* = 6 in each group, * *p* < 0.05, ** *p* < 0.01.

Figure 6 illustrates the distribution and QS of PR in different uterine compartments of rats subjected to different treatments. In the first graph, representing the luminal epithelium, the normal control group shows the highest PR immunostaining, with treatment with testosterone only leading to a significant decrease (*p* < 0.05). The addition of finasteride to the testosterone treatment also leads to a significantly lower QS compared to the control (*p* < 0.05), while the addition of anastrozole and the combination of the two blockers shows a further highly significant decrease (*p* < 0.01).

Pertaining to the glandular epithelium, where a similar pattern to the luminal epithelium can be observed. The control group had the highest PR immunostaining compared to the other treated groups. Although not significant, a decrease in QS was observed in the testosterone treated group. Both the combination of testosterone + finasteride and the combination of testosterone + anastrozole showed a significant decrease in values compared to the control group (*p* < 0.05). After the combination treatment of testosterone + finasteride + anastrozole, a significant decrease in the QS of PR immunostaining in the glandular epithelium was also observed compared to the control group (*p* < 0.01).

However, a different trend can be observed in the PR immunostaining of the stromal compartment where the control group showed a lower QS value compared to the treated groups. In all treated groups, there was a significant increase in the QS score of PR immunostaining compared to the control group (*p* < 0.05).

**Figure 7:**
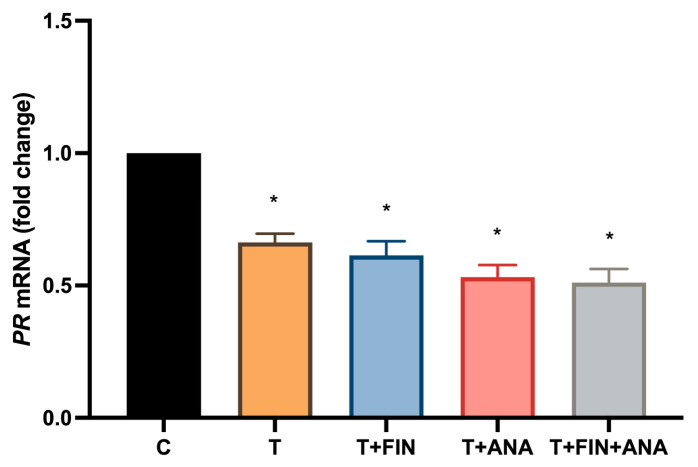
Effects of testosterone on mRNA expression of progesterone receptor (PR). C: normal control; T: testosterone propionate; T + FIN: testosterone propionate + finasteride; T + ANA: testosterone propionate + anastrozole; T + FIN + ANA: testosterone propionate + finasteride + anastrozole. Error bars represent standard error of the mean (SEM). *n* = 6 in each group, * *p* < 0.05 significance compared to normal control group.

Figure 7 shows the PR mRNA expression. In this study, treatment with testosterone leads to a significant reduction in PR mRNA expression (*p* < 0.05) compared to the normal control groups. In addition, all treatments with the combination of testosterone + finasteride, testosterone + anastrozole and the combination of testosterone with both blockers continue to downregulate PR mRNA expression while maintaining statistical significance (*p* < 0.05).

## 3. Discussion

Endometrial receptivity is a brief period of endometrial maturation, that occurs between days 20 and 24 (mid-luteal phase) of a regular menstrual cycle. In rats, however, endometrial receptivity occurs between days 4 and 5 of post-coitus [20,21]. During this period, known as the window of implantation (WOI), the sex steroid hormones play a role in ensuring that the endometrial environment provides the necessary conditions for the implantation of a blastocyst on the surface of the endometrium. This can only be achieved if the endometrium has reached a correct morphological and functional state [22]. Furthermore, it has been shown that the sex steroid hormones, in particular oestrogen and progesterone, are of decisive importance for the establishment of endometrial receptivity, which promotes successful implantation of the embryo [7,23,24].

Oestrogen plays an important role in the initial differentiation of the endometrium, and progesterone promotes the transformation of the endometrium from the proliferative phase to the secretory phase, making the endometrium suitable for implantation and embryo growth. This study has shown that oestrogen and progesterone levels decrease significantly on the evening of postcoital day 4. The decrease in serum oestrogen was significantly greater in the testosterone treated groups compared to the control group. This significant decrease could be due to the impairment of the hypothalamic-pituitary-gonadal (HPG) axis by testosterone. In the present study, co-administration of anastrozole, a non-steroidal aromatase inhibitor, with testosterone leads to a decrease in serum oestrogen compared to the control group due to its inhibitory effect on the aromatase conversion of testosterone to oestrogen, but the decrease is not significant compared to the testosterone-treated group. This is consistent with another study showing that anastrozole also significantly decreases serum oestrogen in the postmenopausal population [25], however, the study on the combination of hyperandrogenic state and anastrozole treatment is only partially comparable. In addition, our results showed that the addition of finasteride, a 5α-reductase inhibitor, to testosterone treatment, although not statistically significant, increased serum oestrogen levels compared to the testosterone treated group without finasteride.

In contrast, our study found that there was a significant decrease in serum progesterone levels in all testosterone-treated groups. It has been shown that hyperandrogenism can interfere with the normal function of the corpus luteum, leading to a decrease in progesterone levels [26]. However, this finding was in contrast to previous studies in which increased testosterone levels caused an increase in serum progesterone levels [27,28]. The addition of an aromatase inhibitor and a 5α-reductase inhibitor increased serum progesterone levels, albeit not significantly, but they were still significantly lower than in the control group. This proves that high testosterone levels together with significantly low oestrogen and progesterone levels have a negative effect on the receptivity of the endometrium, as they lead to altered receptor expression and insufficient development of the endometrium, making it less suitable for embryo implantation [29].

In addition, oestrogen has been shown to stimulate ERα during the proliferative phase, while progesterone inhibits it during the implantation window [30]. The inhibition and reduction of ERα corresponds with endometrial gene expression in the mid-luteal phase, which is a key process for the establishment of endometrial receptivity [31]. Our qPCR analysis showed that the testosterone-treated groups with and without the inhibitors, lead to significantly reduced ERα mRNA expression. Further immunohistochemical analysis of ERα showed significantly reduced distribution of ERα in the glandular epithelium and stroma in all treatment groups, especially in the group with testosterone only and testosterone in combination with both inhibitors. Normally, proliferation of the uterine epithelium in response to oestrogen requires stromal oestrogen receptor alpha (ERα), whereas differentiation of the uterine epithelium requires both epithelial and stromal ERα [32]. In addition, the bioavailability of oestrogen plays a crucial role in determining the receptivity of the endometrium in rodents, implying that insufficient oestrogen levels prevent the transition from the pre-receptive phase to the receptive phase [33]. Our findings are consistent with a previous study that concluded that ERα expression was downregulated in the group receiving a combination treatment of testosterone and oestrogen. In contrast, ERα expression was found to be upregulated in the group receiving oestrogen alone [34]. From our study, downregulation of ERα in both the epithelial and stromal compartments of the endometrium during endometrial receptivity in the presence of supraphysiological testosterone potentially reduces endometrial cell proliferation and differentiation and ultimately leading to a delayed implantation window or implantation failure.

The PR has a dynamic, unique expression pattern in the peri-implantation uterus. It is strongly expressed in the luminal epithelium of the uterus during preimplantation and disappears from it after implantation [35]. In a normal cycle, the secretory endometrium becomes receptive to embryo implantation during the mid-luteal phase. This is initiated by progesterone as it influences the expression of the endometrial transcriptome, which regulates the differentiation of the epithelial and stromal compartments [36,37]. In the present study, supraphysiological testosterone treatment with or without the inhibitors resulted in decreased expression of PR in the luminal and glandular epithelium on the evening of postcoital day 4. In addition, although the expression of PR in the epithelial compartment was downregulated, an increased expression of PR in the stromal compartment was observed. The observed downregulation of PR expression in the luminal and glandular epithelium possibly led to implantation failure. Physiologically, stromal PR mediates the antagonistic activity of progesterone on the proliferative response of the epithelium to oestrogen during the early secretory phase. Nevertheless, an epithelial PR is crucial for stromal and epithelial crosstalk in the uterus. Loss of epithelial PR leads to complete pregnancy failure due to impaired endometrial receptivity.

The level and action of the AR protein, particularly in the uterus, is mainly regulated by the transcription of AR mRNA. With regard to endometrial receptivity, androgen receptor (AR) expression is restricted to the endometrial stroma and fluctuates during the menstrual cycle, progressively decreasing from the early proliferative to the mid-secretory phase [38,39]. Our immunohistochemical study showed that the testosterone-treated groups had significant expression of AR within the endometrial stroma. Moreover, the expression is more pronounced in the testosterone-treated group when additionally treated with anastrozole and/or finasteride. This result is consistent with a previous study reporting that AR protein is mainly found in the nuclei of uterine stromal cells and mouse myometrial cells prior to embryo implantation, but not in the luminal and glandular epithelium. The same finding was observed for AR mRNA expression, with the group treated with testosterone and finasteride even showing higher AR mRNA expression than the testosterone-only group. This could be due to the fact that finasteride has considerable structural similarities with testosterone and DHT. It is therefore possible that finasteride, in addition to its role as a 5α-reductase inhibitor, also acts as a ligand of the AR and alters AR expression in various tissues [40,41].

It has also been reported that AR expression is strongly influenced by progesterone levels with progesterone significantly inhibiting the expression of uterine stromal AR protein [42,43]. Both the AR and PR are able to bind to the same response region and modulate specific gene groups [33]. It is likely that the overexpression of the AR and downregulation of the PR can be attributed to the presence of high testosterone levels. These findings further support the current knowledge that hyperandrogenism reduces fertility by increasing AR expression, which ultimately leads to delayed endometrial decidualisation due to impaired differentiation of endometrial stromal cells [7,44].The modulation of AR by androgen is complex and tissue specific. AR mRNA transcription is downregulated after androgen exposure in prostate, testis, epididymis, seminal vesicle, kidney genital skin fibroblasts, brain and the human prostate cancer cell line, LNCaP [45,46]. In contrast, androgen exposure variably upregulated AR mRNA transcription in the hepatocellular carcinoma cell line, genital skin fibroblasts, rodent prostate, rodent hippocampus and osteoblastic cell line [47,48,49,50,51]. Our results indicate that AR mRNA transcription was upregulated in the uterus after exposure to testosterone, and the upregulation was even more pronounced with the addition of an aromatase inhibitor and a 5α-reductase inhibitor. The up regulation observed in utero after exposure to testosterone suggests a critical role of AR in endometrial receptivity.

We have shown that the effect of testosterone on the expression levels of PR, ERα, level of progesterone and oestrogen is not associated with the enzymatic process of 5α-reductase or aromatase. Our findings showed no difference between all groups treated with testosterone and either finasteride, anastrozole or a combination of both. This suggests that the dysregulation of PR and ERα was not influenced by the presence of DHT or oestrogen, but by supraphysiological exogenous testosterone alone. Our results are consistent with previous studies showing no involvement of DHT in the inhibition of endometrial pinopodes, suggesting that finasteride does not interfere with the testosterone-mediated decrease in pinopode expression [52]. In another study by Gibson et al. (2016), DHT was only detected in endometrial stromal cells (ESCs) that had been stimulated to decidualise [53]. On the other hand, aromatase is not normally present in normal endometrium, but may be transiently present and temporally and spatially regulated during decidualisation [33]. However, according to our results, it is very likely that decidualisation can be disturbed by the deviation of ovarian steroid hormones from supraphysiological testosterone during endometrial receptivity.

Our study has shown that hyperandrogenism leads to significantly lower levels of oestrogen and progesterone (vital sex hormones during the transition to the receptive endometrium). Furthermore, our study highlights that hyperandrogenism disrupts endometrial receptivity by altering AR mRNA and protein expression, potentially leading to impaired decidualisation and delayed stromal cell differentiation.

## 4. Materials and Methods

### 4.1. Animal Preparation and Hormonal Treatment

Adult female Sprague–Dawley rats (*n* = 30) were obtained from the Laboratory Animal Resources Unit, Faculty of Medicine, Universiti Kebangsaan Malaysia. The acquired rats weighed 225 ± 25 g and had at least two consecutive regular cycles. The rats were housed in a controlled environment, with light from 06:00 to 18:00 and a room temperature of 24 ± 2 °C, with one animal per cage. The rats were fed a diet from Harlan, Germany, and had unlimited access to tap water. The experimental procedures were approved by the Animal Ethics Committee of the National University of Malaysia (approval number: FISIO/PP/2019/MOHD HELMY/30-OCT./1060-OCT.-2019-AUG.—2022).

In this study, female rats in the proestrus stage were mated overnight with a male rat of the same species in a 1:1 ratio. The next morning, successful mating was confirmed by microscopic analysis of vaginal swabs for the presence of sperm and the appearance of a vaginal plug (designated as post-coital day 1) [54,55]. The rats were randomly divided into the following groups (*n* = 6 rats per group).

Group 1: Normal rats(C) given peanut oil (vehicle).

Group 2: Rats treated with 1 mg/kg/day testosterone propionate (T) [56].

Group 3: Rats treated with 1 mg/kg/day testosterone propionate and 1 mg/kg/day finasteride (T + FIN) [57].

Group 4: Rats treated with 1 mg/kg/day testosterone propionate and 1 mg/kg/day anastrozole (T + ANA) [57].

Group 5: Rats treated with 1 mg/kg/day testosterone propionate and 1 mg/kg/day finasteride and 1 mg/kg/day of anastrozole (T + FIN + ANA).

All rats were treated for 3 consecutive days, starting from day 1 to day 3 of gestation (early gestation period) [58]. Testosterone propionate, finasteride and anastrozole (Sigma-Aldrich, St Louis, MO, USA) were dissolved in 0.1 mL of peanut oil prior to subcutaneous injection behind the neck scruff. The inhibitory drugs, finasteride and anastrozole, were injected 30 min prior to the injection of testosterone propionate [56]. The supraphysiological dose of testosterone propionate used in this study was selected based on the dose previously used in other studies [57]. The rats were euthanised on the evening of the fourth day of postcoital [56] which is considered the day of endometrial receptivity in rats, by intravenous injection of high-dose ketamine-xylazine at a dose of 0.3 mL per 100 g of body weight. The uteri were then harvested to analyse changes in gene expression and the distribution of AR, ERα and PR.

### 4.2. Determination of Serum Hormone Levels by Enzyme-Linked Immunosorbent Assay (ELISA) Technique

Blood samples were collected by cardiac puncture and serum was collected using serum separator tubes (BD Vacutainer SSTTM, Becton Dickinson, Franklin Lakes, NJ, USA). Samples were allowed to coagulate at a temperature of 4 °C before being spun in a centrifuge at a force of 3000× *g* for 15 min. They were then stored at a temperature of −80 °C until hormonal analysis was performed. Serum hormone levels were measured using a competitive enzyme-linked immunosorbent assay (ELISA) for oestradiol, progesterone and testosterone. Samples were tested in triplicate as recommended by the manufacturer (Elabscience, Houston, TX, USA). A biotinylated antibody solution was combined with reference standards to generate reference wells. The test wells were supplemented with a solution of biotinylated antibodies and serum samples. The plate was then sealed and placed in an incubator at a temperature of 37 °C for 45 min. The plate was cleaned with a wash buffer and repeated a total of three times. Horseradish peroxidase (HRP) conjugate was added to each well and the plate was then incubated at a temperature of 37 °C for 30 min. The plate was then washed a total of five times. A substrate reagent was then added to each well and the plate was incubated at a temperature of 37 °C for 15 min. A stop solution was then added to each well to stop the reaction, and the absorbance of the solution in the wells was immediately quantified using a microplate reader at 450 nm. The standard curve was generated using standard dilutions with known hormone concentrations. The generated standard curve was used to measure the concentrations of serum hormones. 

### 4.3. Histological Examination and Immunohistochemical Staining

All uteri were immersed overnight in a solution of neutral buffered formaldehyde for fixation, followed by a series of ethanol solutions of increasing concentration for dehydration. The uteri were then chemically processed and embedded in paraffin wax. Sections with a thickness of 5 mm were prepared. The slides were then treated with pH 6.0 and pH 9.0 solution of Dako’s antigen retrieval solution (Glostrup, Denmark). To inhibit the activity of endogenous peroxidase, the slides were treated with hydrogen peroxide. The samples were then blocked with the blocking serum from the Mouse and Rabbit Specific HRP/DAB IHC Detection Kit—Micro-polymer (Cat ab236466, Abcam, Cambridge, MA, USA). To investigate the distribution of the sex steroid receptor proteins, the slides were incubated for 1 h at room temperature with a rabbit polyclonal antibody against the progesterone receptor at a dilution of 1:400 (Cat. AB11370 Abcam) or a rabbit antibody against the oestrogen receptor alpha at a dilution of 1:200 (Cat. AB65969 Abcam) and a rabbit antibody against the androgen receptor at a dilution of 1:200 (Cat. AB65969 Abcam). The slides were then treated with the micropolymeric secondary antibody from the Mouse and Rabbit Specific HRP/DAB IHC Detection Kit (Cat ab236466, Abcam). To identify the protein, the slides were treated with the DAB substrate from the Mouse and Rabbit Specific HRP/DAB IHC Detection Kit-Micro-polymer (catalogue number ab236466, Abcam). Finally, the sections were stained with haematoxylin and then dehydrated sequentially. Each of the slides was examined using an Olympus BX53 light microscope (Olympus Corporation, Tokyo, Japan). The stained samples were then observed and imaged using an Olympus DP27 colour camera system (Japan). All images were taken with precisely the same parameters for all experimental groups. Immunohistochemistry was assessed semi-quantitatively using the Quickscore with a cut-off positivity value of ≥3 [59,60]. Immunostaining analyses were performed by two observers (an anatomic pathologist and a doctorate student), at least one of whom did not know the classification of the experimental group. In case of disagreement, the analyses were reviewed jointly, and consensus was reached. In this study, the widely used combine semi-quantitative scoring Quickscore system was used, which provides for an intensity score of 0–3 and a ratio score of score 0–5. Scores of 0 and 2 are considered negative. Scores of 3 to 8 are considered positive [61]. Table 1 illustrates the details of the QS scoring.

### 4.4. RNA Isolation and Quantitative Polymerase Chain Reaction (qPCR)

The preserved rat uteri were fixed in RNA Later solution (Sigma Aldrich, Saint Louis, MO, USA) to stabilise and maintain RNA integrity in the cells. The Nucleospin RNA isolation kit (CAT 740955.50 Macherey-Nagel, Duren, Germany) was used for RNA extraction from the uterus. The RNA extraction procedure was performed according to the manufacturer’s guidelines. The absorbance of each sample was quantified at wavelengths of 260 nm and 280 nm and the purity of the RNA was determined using the 260/280 ratio (Gene Quant 1300, Cambridge, UK). Conversion of RNA to complementary DNA (cDNA) was performed using the qPCRBio cDNA synthesis kit (CAT PB30.11-10 PCR Biosystems, London, UK). Amplification of samples without the addition of reverse transcriptase (-RT) served as a control. The qPCR master mix was prepared using the qPCRBio SyGreen Blue Mix Kit (CAT PB20.17-05 PCR Biosystems). The reference gene used in this study was glyceraldehyde-3-phosphate dehydrogenase (GAPDH). Specific primers for androgen receptor (catalogue number: RQP129826, ID: Rn-QRP-10084, GeneCopoeia, Rockville, MD, USA), oestrogen receptor-α, (catalogue number: RQP049019, ID: Rn-QRP-12024, GeneCopoeia), progesterone receptor (catalogue number: RQP050618, ID: Rn-QRP-12441, GeneCopoeia) and glyceraldehyde-3-phosphate dehydrogenase (GAPDH) (catalogue number: RQP049537, ID: Rn-QRP-10195, GeneCopoeia) were used for qPCR. The BioRad CFX96 Real-Time System was used to perform real-time PCR. The conditions were as indicated: The polymerase is activated by incubation at 95 °C for 5 min. This is followed by denaturation, where the sample is left at 95 °C for 10 s, which is repeated for 40 cycles. Adhesion and elongation are performed at 60 °C for 30 s, which is also repeated for 40 cycles. The experiments were performed in triplicate. Data were analysed using the comparative CT (2-∆∆Ct) method. The relative amount of each amplicon was determined by comparing the normalised amount of each gene with the normalised amount of each reference gene.

### 4.5. Statistical Analysis

The data were analysed using parametric analysis of variance (ANOVA) followed by a post-hoc test using the Dunn test. The Shapiro-Wilk normality test was used to test the normality of the data. If the data distribution was abnormal, the Kruskal-Wallis test was performed. The difference was considered statistically significant if *p* < 0.05. GraphPad Prism version 10.2.3 (347) was used for statistical analysis in this study.

## 5. Conclusions

This study shows that supraphysiological dose of testosterone may be able to interrupt the short period of the implantation window. Overall, our study sheds light on the intricate interplay between androgens and sex steroid hormones in the regulation of endometrial receptivity development and emphasises the detrimental effects of hyperandrogenism on fertility through its influence on AR, ERα and PR expression patterns. These findings could serve as a basis for understanding the role of testosterone in endometrial receptivity to develop further therapeutic approaches targeting androgen-mediated disorders of endometrial receptivity. However, the present study has the limitation that neither the number of implantation sites nor other potential pathways that could affect endometrial receptivity were investigated to confirm the effect of supraphysiological testosterone. Therefore, a further study is required to investigate the effects of supraphysiological testosterone on the potential molecular signalling pathways involved in endometrial receptivity, the decidualisation process and implantation of the embryo.

## Figures and Tables

**Table 1:** Quickscore system.

Proportion Score	% of Positive Cells	Intensity	Intensity Score
0	0	None	0
1	<1	Weak	1
2	1 to 10	Intermediate	2
3	11 to 33	Strong	3
4	34 to 66	QS (proportion score + intensity score): 0–8	
5	>67		

## Data Availability

The original contributions presented in the study are included in the article, further inquiries can be directed to the corresponding author.
